# Michigan State Board of Health

**Published:** 1880-06

**Authors:** 


### ARTICLE VI.

### THE MICHIGAN STATE BOARD OF HEALTH.

The regular quarterly meeting of the State Board of Health was held in Lansing on April 13. The members present were Dr. H. O. Hitchcock, of Kalamazoo; Leroy Parker, of Flint; Rev. D. C. Jacokes, of Pontiac; Dr. J. H. Kellogg, of Battle Creek, and H. B. Baker, secretary.

The president, Dr. Kedzie, being absent, Dr. Hitchcock was chosen president *pro tem.*

The secretary presented a letter from Dr. Kedzie, stating that the severe illness of his son, Prof. W. H. Kedzie, would prevent him from attending the meeting. In this connection the board adopted a resolution expressing sincere sympathy for Dr. Kedzie and family.

WORK IN THE OFFICE.

The secretary read the quarterly reports of work in the office for the quarters ending January 7 and April 13 respectively. After the meeting in October, 1879, a large number of copies of the Lansing *Republican* containing an abstract of the proceedings of the meeting and the complete report of the committee on sanitary conventions was sent out to manufacturers and dealers in sanitary appliances, and to sanitarians, in order to secure as large exhibits and attendance as possible. Circulars of the sanitary conventions at Detroit and Grand Rapids were also sent out from the office. Sixteen regular correspondents in the State have been added to the list. Mortality statements for the city of Lansing have been sent for each month to a large number of exchanges. The record of meteorological observations taken at the office and printed in the *Republican* are reprinted and sent weekly to about thirty-five observers of the board and contributors of meteorological data. One hundred and thirty-five monthly meteorological registers have been received from observers. The card catalogue system of keeping the library in the office has been adopted. The library numbers over 2,000 accessions. Daily papers containing accounts of the sanitary conventions at Detroit and Grand Rapids have been sent from the office quite freely. The secretary has completed a paper on "Glanders in Man and Animals" for the forthcoming annual report, and a paper on "General Sanitation," which was read at Detroit. There have been distributed from this office, 5,215 copies of circular 34, 6,258 of circular 35, and 3,129 of the document on the "Restriction and Prevention of Diphtheria."

Of circulars 36 and 37, transmitting blanks for annual reports of clerks and health officers, there have been sent of each, 1,369 copies. Annual reports have been received from about 575 clerks and 550 health officers. A circular to supervisors of townships, asking for a return of name and address of health officer for ensuing year, has been sent out to the 1,440 townships. Copies of circular 38, relative to diseases in Michigan, 1879, have been sent to 143 regular correspondents. Circular 39, asking for the return of name and address of health officers of cities and villages, has been sent to 440 clerks, mayors and presidents. Blank meteorological registers to the number of 1,134 have been sent to observers; and 2,495 postal card blanks sent to observers of diseases. The compilation of material for the report for 1880 has gone steadily on. In the correspondence of the office 668 pages of the letter book have been used. New meteorological stations have been established at Hillsdale and Marshall, and arrangements started for two more—at Onondaga and Reed City. Of the communications received, 45 have been referred to committees of the board.

The secretary presented some documents issued by the local board of health of Tecumseh, as illustrative of what a live, energetic board of health might accomplish.

Mention was also made of the health officers and authorities of Lansing, who have done good sanitary work, and succeeded in establishing a system for the collection and registration of vital statistics, which requires burial permits, Lansing being the first city in the State to take this commendable step. Muskegon, under the lead of Mayor Holt, was also mentioned for active efforts for the prevention of disease.

A communication from C. H. Voute of East Saginaw, stated that he desired to form a circuit of towns and cities in this State, for using the odorless excavating apparatus for the removal of contents of privy vaults. A resolution was adopted recommending local boards of health to secure the cleaning of vaults by means of such apparatus, wherever the dry earth system is not in use.

The present editions of the documents on the Restriction and Prevention of Scarlet Fever, and on the Restriction and Prevention of Diphtheria, being practically exhausted, it was decided to have them revised, published in the next annual report, electrotyped, and a large edition of each document printed. As it is to be electrotyped, local boards of health may procure any number of either document at a slight cost.

### DIPHTHERIA.

The secretary stated that, inasmuch as diphtheria has been so prevalent in this State, it has been suggested by an officer of the national board of health that this was a favorable field for a systematic investigation of the causes of the disease, particularly as to what are its relations, if any, to filth. The subject was thoroughly discussed, at some length, and the great desirability of such an investigation was unanimously conceded, but the resources of the board are entirely inadequate for such a house to house inspection as seems essential.

The secretary was directed to correspond with the national board of health and see what arrangements can be made for such an investigation.

### SANITARY CONVENTIONS.

The secretary was authorized to begin printing the proceedings of the recent sanitary conventions at Detroit and Grand Rapids as soon as practicable. The report of the board for 1879 is now in press, and will shortly be issued.

Dr. Kellogg, as committee on the disposal of decomposing organic matter, presented a paper on

### DECAYING WOOD A CAUSE OF DISEASE.

He related experiments by Prof. Wm. H. Brewer, confirmed by himself, showing that when green wood was allowed to stand for some time in water the solution decomposes, and gives off very offensive odors. Even when the water was renewed again and again, the similar result ensued. The paper was prepared with special reference to the practice of putting sawdust in streams and ponds, and it tended to confirm the belief that the practice is frequently productive of malarial and diarrhœal diseases.

### SANITARY SURVEY.

Dr. Jacokes, chairman of the committee on such survey, made a statement relative to the desirability of having a sanitary survey of the State, and as to its probable extent and cost.

SANITARY SCIENCE EXAMINATIONS.

July 14, the day after the next meeting of the board, it will, if candidates apply, examine them in sanitary science, giving a certificate of merit to those who pass a satisfactory examination. An outline of the plan of these examinations will appear in the forthcoming report for 1879.

An unusual amount of routine business was transacted, auditing of accounts, etc. The next meeting of the board will be July 13.

